# Short-term ketamine interventions for depressive disorders: practical best-practice recommendations for psychiatrists using oral, subcutaneous, and nasal formulations

**DOI:** 10.3389/fpsyt.2026.1931248

**Published:** 2026-09-03

**Authors:** Anita Słupska, Wiesław Jerzy Cubała, Damian Swieczkowski, Joanna Szarmach, Jakub Słupski, Adam Włodarczyk

**Affiliations:** 1 Department of Psychiatry, Faculty of Medicine, Medical University of Gdańsk, Gdańsk, Poland; 2 Department of Exercise Physiology and Functional Anatomy, Ludwik Rydygier Collegium Medicum in Bydgoszcz, Nicolaus Copernicus University in Toruń, Bydgoszcz, Poland

**Keywords:** esketamine, intranasal ketamine, ketamine, off-label prescribing, oral ketamine, psychiatric practice, subcutaneous ketamine, treatment-resistant depression

## Abstract

**Objectives:**

Ketamine and esketamine are increasingly used for treatment-resistant depressive disorders, particularly when rapid symptom reduction is clinically desirable. Intranasal esketamine has a product-specific regulatory framework in several jurisdictions, whereas most racemic ketamine formulations remain off-label for psychiatric indications. This narrative review provides practical recommendations for psychiatrists considering short-term ketamine interventions in depressive disorders, with emphasis on oral solution, oral tablets, subcutaneous injection, and nasal spray formulations.

**Methods:**

We conducted a narrative review and clinical-practice synthesis of PubMed/MEDLINE-indexed literature, open bibliographic sources, and regulatory documents, searched through 20 July 2026. Search concepts covered ketamine and esketamine, treatment-resistant major depressive disorder, intravenous, oral, subcutaneous, and intranasal administration, dosing, monitoring, treatment setting, substance-use risk, misuse/diversion, and short-term intervention. Evidence was prioritized by study design and regulatory status; practical recommendations were categorized according to whether they were label-supported, directly evidence-supported, or based mainly on expert-practice synthesis.

**Results:**

Evidence is strongest for labeled intranasal esketamine and intravenous racemic ketamine. Among the non-IV approaches reviewed here, subcutaneous racemic ketamine has coherent randomized evidence for flexible-dose short-term treatment; oral ketamine has limited but supportive small-trial and systematic-review evidence, with newer prolonged-release tablets remaining formulation-specific and investigational; and compounded racemic intranasal ketamine has weaker, more heterogeneous evidence. For all off-label racemic formulations, optimal dosing, frequency, durability, long-term safety, misuse risk, and maintenance strategy remain incompletely established.

**Conclusions:**

Short-term ketamine treatment should be protocolized, measurement-based, supervised, time-limited, and embedded in a broader treatment plan for depressive disorders. For off-label racemic ketamine, the best-supported non-IV short-term regimen is supervised subcutaneous dosing with response-guided titration. Oral ketamine may be considered when parenteral or regulated nasal options are inaccessible, but it requires cautious patient selection, controlled dispensing, and objective monitoring. Esketamine nasal spray should follow the approved product label where available, whereas compounded racemic nasal ketamine should be reserved for exceptional circumstances.

## Highlights

- Diagnose before dosing. Confirm the depressive disorder, treatment resistance, bipolarity risk, psychosis risk, substance-use history, cardiovascular risk, and medical contributors to depression.
- Use a short-term treatment contract. Define the formulation, dose, frequency, monitoring schedule, permitted dose escalations, and stopping rules before the first treatment.
- Prefer supervised administration. All first doses and all dose increases should occur under direct clinical supervision. Less-supervised treatment should be exceptional and restricted to carefully selected patients.
- Titrate to benefit, not dissociation. The clinical goal is meaningful antidepressant benefit with the lowest effective exposure, not a dissociative experience.
- Measure response. Use a depression scale, global clinical impression, suicide-risk assessment, and adverse-event log before each treatment.
- Stop early when ineffective or unsafe. Discontinue after 4–6 administrations when there is no objective improvement, and stop immediately for mania, psychosis, sustained hypertension, serious dissociation, misuse signals, or urinary toxicity.
- Govern controlled-substance risk. Use limited dispensing, prescription-monitoring checks where available, no early refills, locked-storage counseling, and documentation of craving, drug liking, and diversion risk.

## 1 Introduction

Ketamine is a racemic mixture of R- and S-enantiomers; esketamine is the S-enantiomer alone. Within psychiatry, the firmest evidence concerns intravenous racemic ketamine and intranasal esketamine, both of which can produce rapid antidepressant effects in some patients with treatment-resistant depression (TRD). Walsh et al. (1) noted that these antidepressant and anti-suicidal effects, though often rapid, can also be short-lived, and McIntyre et al. (2) stressed that translating them into practice depends on careful attention to diagnosis, route of administration, monitoring, durability, and risk governance.

Implementation, however, remains uneven. Access, cost, regulatory frameworks, staffing, monitoring capacity, and patient preferences differ widely from one setting to another. Kasper et al. (3) framed esketamine treatment not as a simple prescription but as a protocolized intervention that folds evidence, patient selection, dosing, observation, and follow-up into a single workflow. That framing matters most when psychiatrists reach for off-label racemic ketamine formulations, which carry no product-specific psychiatric labeling (4, 5).

Our focus here is short-term intervention rather than open-ended maintenance. By short-term intervention we mean a structured course of roughly 2–4 weeks intended to achieve rapid symptom reduction, gauge treatment response, and hand the patient on to evidence-based continuation care. Such a course may be especially useful in treatment-resistant major depression and in severe depressive episodes with marked functional impairment.

The line between regulated esketamine and off-label racemic ketamine is clinically consequential. Esketamine nasal spray comes with its own dosing, administration, and monitoring requirements. Under the current U.S. prescribing information, Janssen Pharmaceuticals, Inc. (6) indicates esketamine for TRD in adults, either as monotherapy or with an oral antidepressant, and for depressive symptoms in adults with major depressive disorder (MDD) and acute suicidal ideation or behavior in combination with an oral antidepressant. Racemic ketamine given orally, subcutaneously, or as a compounded nasal product is generally off-label for depressive disorders and lacks standardized labeling for psychiatric use. Accordingly, the aim of this review is to provide formulation-specific, evidence-based, and clinically relevant recommendations for short-term ketamine and esketamine administration in depressive disorders.

## 2 Methods

This is a narrative clinical review with best-practice recommendations for adult psychiatrists. Its structure follows the practical-recommendation approach of Kasper et al. (3), who combined basic science, trial evidence, and expert guidance for managing TRD with esketamine nasal spray therapy.

We reviewed the available evidence through PubMed/MEDLINE-indexed literature, open bibliographic sources, and regulatory documents. Search terms included ketamine, esketamine, racemic ketamine, treatment-resistant depression, major depressive disorder, suicidal ideation, intravenous ketamine, oral ketamine, ketamine tablets, extended-release ketamine, prolonged-release ketamine, subcutaneous ketamine, intranasal ketamine, nasal spray, dose, frequency, monitoring, treatment setting, dissociation, blood pressure, substance-use disorder (SUD), abuse, misuse, and diversion. We gave priority to randomized controlled trials, systematic reviews, product labeling, regulatory safety communications, consensus statements, and clinically oriented guidance.

Each bibliographic entry was checked against PubMed records, journal and publisher websites, official prescribing information, and regulatory documents. Claims about formulation, route, dose, or regulatory status were phrased conservatively to avoid overgeneralization. Findings on investigational prolonged-release oral ketamine, for instance, are reported as formulation-specific and are not carried over to justify dose conversion for compounded oral ketamine.

The search was last updated on 20 July 2026. Because this was a narrative rather than systematic review, study identification was iterative and purposive rather than based on a PRISMA flow. Eligible sources included adult studies of ketamine or esketamine for depressive episodes, systematic reviews, consensus or practice guidance, and official regulatory or product documents that directly informed route-specific efficacy, safety, monitoring, treatment-setting, or misuse-governance statements. We excluded pediatric-only reports, non-depressive primary indications, preclinical studies, articles without route- or practice-relevant information, duplicate reports, and sources whose formulation could not be linked reliably to the recommendation under discussion. Twenty-nine core publications or regulatory documents were retained in the final synthesis. The complete PubMed/MEDLINE search string is provided in **Supplementary Material 1**.

Evidence conflicts were handled by privileging approved product information for labeled use, then systematic reviews and randomized trials, followed by prospective or retrospective observational evidence. When findings differed across formulations or settings, they were not pooled conceptually; the manuscript reports the disagreement and limits the recommendation to the population, route, formulation, and duration directly studied.

Recommendations were drafted from the evidence synthesis and revised through iterative review by all authors. All final recommendations were approved unanimously, and disagreements were resolved by discussion without formal voting. No recommendation was strengthened solely because of an author’s clinical experience or industry relationship; where direct evidence was insufficient, the wording is identified as an expert-practice proposal rather than an evidence-based protocol.

For transparency, recommendations are grouped into three categories: Category A, label-supported or supported by multiple randomized trials/systematic reviews; Category B, supported by at least one directly relevant randomized trial or a consistent body of observational evidence; and Category C, expert-practice proposals or cautious extrapolations used when direct evidence is sparse. These categories describe the evidentiary basis of this narrative synthesis and are not a formal GRADE assessment (**Table 1**).

**Table 1:** Evidentiary basis of selected practical recommendations.

Recommendation	Category	Basis	Interpretation
Use labeled intranasal esketamine according to product information	A	Regulatory label and multiple randomized controlled trials (RCTs)	Evidence-based, product-specific
IV racemic ketamine 0.5 mg/kg over about 40 minutes as reference regimen	A	Multiple RCTs, systematic reviews, consensus guidance	Evidence-based reference standard; off-label
Flexible-dose subcutaneous ketamine 0.5-0.9 mg/kg twice weekly	B	Direct randomized KADS evidence	Evidence-informed, short-term, supervised
Repeated oral racemic ketamine solution	B/C	One small RCT plus heterogeneous reviews	Possible option; protocol details partly expert-derived
Compounded racemic intranasal ketamine	C	Sparse heterogeneous evidence	Exceptional use only
Setting, prescriber, stopping, and diversion safeguards	B/C	Regulatory/consensus guidance plus conservative expert practice	Operational safeguards; not all tested in RCTs
Ketamine with current or past SUD	C	Small real-world samples; pivotal trials often excluded active SUD	Individualized specialist decision with enhanced monitoring
Forensic or prison settings	C	Ethical/legal analysis; minimal outcome data	Exceptional, directly observed specialist treatment

Generative AI tools were used only for language and stylistic editing, reference-format consistency checks, and document preparation; they were not used to select studies, interpret evidence, or formulate recommendations.

## 3 Scope and clinical target population

These recommendations are written for adult psychiatric practice and for psychiatrists managing depressive disorders in structured clinical settings. The core target population is adults with TRD, usually defined as an inadequate response to at least two adequate antidepressant trials in the current episode. Janik et al. (7), for example, defined eligibility in their phase 4 esketamine monotherapy trial as adults with DSM-5 major depressive disorder without psychotic features who had responded inadequately to at least two oral antidepressants. Bipolar depression is outside the scope of this article and will be addressed separately.

## 4 General principles for dose selection

Dose selection should be individualized around four variables: clinical urgency, prior ketamine exposure, medical risk, and route-specific pharmacokinetics. Andrade (8) cautioned that oral ketamine has lower and more variable bioavailability than the parenteral routes, which makes any simple dose equivalence with intravenous regimens unsafe. For intranasal esketamine, Janssen Pharmaceuticals, Inc. (6) reports a bioavailability of roughly 48% and a median time to peak plasma concentration of about 20–40 minutes after the last spray in a session.

The guiding principle is to begin low to protect safety, titrate only when benefit is inadequate and tolerability is good, and stop early when there is no response. The target is a clinically meaningful antidepressant effect at the lowest effective exposure, not the induction of dissociation. Escalation should be held back if the patient develops severe dissociation, clinically significant hypertension, prolonged sedation, agitation, psychotic or manic symptoms, craving, drug-liking behavior, or repeatedly asks for unsupervised dose increases.

A sound short-term course rests on baseline symptom measurement and repeated assessment before each treatment. Useful instruments include the Montgomery-Åsberg Depression Rating Scale (MADRS), the Quick Inventory of Depressive Symptomatology (QIDS-SR), the Patient Health Questionnaire-9 (PHQ-9), the Clinical Global Impression scales (CGI), the Columbia Suicide Severity Rating Scale (C-SSRS), the Young Mania Rating Scale (YMRS) where relevant, the Clinician-Administered Dissociative States Scale (CADSS) where available, and a structured adverse-event checklist.

## 5 Formulation-specific dosing and administration

An evidence-informed hierarchy of routes is summarized in **Table 2**, and practical short-term dosing recommendations are presented in **Table 3**.

**Table 2:** Evidence-informed route hierarchy for short-term ketamine interventions in depressive disorders.

Route/formulation	Evidence position	Recommended place in practice
Esketamine nasal spray	A regulated product with its own dosing, observation, and risk-mitigation requirements. The evidence spans acute randomized trials, relapse-prevention data, and more recent randomized monotherapy data (9, 7, 6, 10).	Use according to the approved label in jurisdictions where it is available and clinically appropriate. Do not substitute compounded racemic nasal ketamine protocols for labeled esketamine protocols.
Subcutaneous racemic ketamine	The most coherent off-label, non-intravenous racemic dosing model. In a 4-week active-controlled trial, Loo et al. (11) found flexible, response-guided dosing from 0.5 to 0.9 mg/kg more convincing than fixed 0.5 mg/kg dosing.	The preferred off-label, non-intravenous racemic route where supervised clinic dosing and monitoring are feasible.
Oral racemic ketamine solution	Limited but supportive controlled evidence. Domany et al. (12) used 1 mg/kg three times weekly for 21 days, while systematic reviews stress heterogeneity and limited certainty (13, 14).	Consider only with careful consent, a supervised first dose and supervised escalations, controlled dispensing, and strict stopping rules.
Oral tablets, capsules, troches, lozenges	Formulation-dependent evidence. Immediate-release compounded products are not interchangeable with investigational extended- or prolonged-release tablets (15, 16).	Use cautiously, documenting the product, compounding pharmacy, release characteristics, dose accuracy, and dispensing controls.
Compounded racemic nasal ketamine	Weaker and more heterogeneous evidence. An et al. (17) included only one racemic ketamine randomized trial among mostly esketamine studies, and Boudieu et al. (18) stressed the need for more route-specific evidence.	Reserve for exceptional specialist settings, where better-supported routes are unavailable and safeguards are strong.

**Table 3:** Practical short-term dosing recommendations.

Formulation	Dose selection and frequency	Administration safeguards
Oral racemic ketamine solution	**Dose:** 0.5–1.0 mg/kg orally under observation. **Titration:** increase by 0.25–0.5 mg/kg only when the dose is tolerated and the response inadequate. Reported regimens have used roughly 1–2 mg/kg, but schedules vary widely across studies. **Frequency:** 2–3 times weekly for 2–3 weeks; reassess after 4–6 doses; avoid routine daily use outside research.	Prefer a pharmacy-compounded solution of verified concentration. Observe the first dose and every increase, and dispense minimal quantities. Document mood, blood pressure, dissociation, sedation, nausea, urinary symptoms, and any misuse signals.
Oral immediate-release tablets, capsules, troches, lozenges	**Dose:** apply the same cautious principles as for the oral solution, but do not assume mg-for-mg equivalence across products. **Titration:** individualize, and avoid extrapolating from extended-release trials to compounded products. **Frequency:** generally, no more than 2–3 administrations weekly during acute evaluation.	Use only where product quality, dose accuracy, and dispensing control are assured. Avoid bulk home supplies, and require a treatment agreement, no early refills, and symptom monitoring.
Investigational extended- or prolonged-release ketamine tablets	**Evidence:** formulation-specific. Glue et al. (15) used R-107 at 120 mg daily for 5 days as open-label enrichment, then randomized responders to 30, 60, 120, or 180 mg, or placebo, twice weekly. Walter et al. (16) studied KET01 at 120 or 240 mg/day for 3 weeks; the primary day-21 efficacy endpoint was not met, though early exploratory signals were seen. **Comment:** these data are not conversion guides for compounded oral ketamine.	Use only within trials or authorized settings. Do not translate trial doses mg-for-mg to compounded immediate-release tablets, capsules, or troches.
Subcutaneous racemic ketamine	**Dose:** 0.5 mg/kg subcutaneously. **Titration:** if the response is inadequate and tolerability acceptable, titrate in approximately 0.1 mg/kg increments toward 0.9 mg/kg. **Frequency:** twice weekly for 4 weeks; reassess after dose 4 and dose 8.	Supervised clinic dosing. Monitor blood pressure, pulse, mental status, dissociation, sedation, nausea, anxiety, and psychotomimetic effects. Observe for at least 2 hours, especially during initiation and escalation.
Esketamine nasal spray	**Dose (TRD, adults):** 56 mg or 84 mg twice weekly during weeks 1–4 after the first treatment decision; 56 mg or 84 mg weekly during weeks 5–8; then weekly or every 2 weeks, using the least frequent dosing that maintains response. Under the current U.S. label, TRD may be treated as monotherapy or with an oral antidepressant. **MDD with acute suicidal ideation/behavior**: 84 mg twice weekly for 4 weeks, reduced to 56 mg if needed, alongside an oral antidepressant.	Follow the product label. Each device delivers 28 mg, so 56 mg requires two devices and 84 mg three. Allow a 5-minute rest between devices, monitor for at least 2 hours, and advise no driving until the next day after restful sleep.
Compounded racemic nasal ketamine	**Dose:** 25–50 mg total intranasal observed dose, delivered in divided metered sprays. **Titration:** in experienced settings, consider cautious titration to a total of 50–100 mg only if tolerated and clinically justified. **Frequency:** once or twice weekly during a short trial; stop if there is no meaningful benefit after 2–4 administrations.	Not equivalent to esketamine. Avoid home bottles during initiation. Use metered, supervised dosing, and document spray concentration, number of sprays, nostril sequence, and timing. Monitor as for esketamine.

Boldface is used to identify descriptive subheadings within the table (e.g., Dose, Titration, Frequency, Evidence, and Comment) and does not indicate statistical significance or a higher level of evidence.

### 5.1 Intravenous racemic ketamine as the reference standard

Intravenous racemic ketamine was not the primary operational focus of this review because its administration, staffing, and monitoring requirements have been addressed extensively in prior consensus guidance. It is nevertheless included here as the comparative reference standard requested by the reviewer. The most established short-term regimen is 0.5 mg/kg infused over approximately 40 minutes, usually once or twice weekly, with direct medical supervision and physiological and mental-status monitoring (2, 19, 20). Its advantages are complete bioavailability, precise dose delivery, rapid onset, and the largest clinical evidence base for racemic ketamine. Its disadvantages are the need for infusion facilities, trained staff, venous access, post-dose observation, and a higher service burden than subcutaneous or oral treatment.

In acute inpatient care, intravenous ketamine may be incorporated into an existing electroconvulsive therapy (ECT), anesthesia, or medically supported procedure pathway. A naturalistic inpatient series found that repeated 0.5 mg/kg infusions could be delivered within psychiatric hospitals, including an ECT suite or post-anesthesia care unit, but also documented treatment-contemporaneous dysphoria and the need for clear escalation pathways (21). IV ketamine should therefore be treated as a benchmark for efficacy and monitoring, not as a dosing template that can be converted directly to oral, subcutaneous, or compounded intranasal formulations.

A 2025 retrospective study of 30 patients with TRD compared concurrent ECT and intravenous racemic ketamine, administered on consecutive days, with ECT alone. Mean reductions in Hamilton Depression Rating Scale scores were similar in the two groups (67.31% and 67.22%, respectively), with no significant between-group difference. The authors proposed the combined approach as an option for patients who had not responded to ketamine or ECT used separately, although the small sample and retrospective design limit the strength of inference (22).

### 5.2 Oral racemic ketamine solution

Oral ketamine is attractive because it is inexpensive and noninvasive, but its bioavailability is lower and more variable than that of parenteral formulations, and the supporting data are thinner. Domany et al. (12) ran a randomized, double-blind, placebo-controlled proof-of-concept study in 41 outpatients with TRD, giving either 1 mg/kg oral ketamine or placebo three times weekly for 21 days. Depressive symptoms fell more with ketamine, and remission occurred in 27.3% of ketamine-treated participants versus none on placebo. The signal is clinically relevant but should be read cautiously: it comes from a single small trial rather than an established dosing regimen.

Rosenblat et al. (14) judged the early oral-ketamine findings to show antidepressant effects with generally acceptable tolerability, while cautioning that responses looked slower and the evidence less definitive than in the intravenous literature. Updating that picture, Meshkat et al. (13) again found the studies heterogeneous, differing in formulation, dosing schedule, enantiomer, co-treatment, depression subtype, and follow-up. These differences are not academic: oral absorption, first-pass metabolism, and metabolite exposure can all shape efficacy and tolerability.

When oral treatment is chosen for short-term off-label use, an oral solution is preferable to nonstandard tablets, because the concentration can be specified and the dose adjusted precisely. A reasonable observed starting dose is 0.5-1.0 mg/kg, lowered further for older adults, medically fragile patients, those anxious about dissociation, or those on sedating co-medications. If the first dose is well tolerated but ineffective, subsequent sessions can rise by 0.25-0.5 mg/kg. As a rule, the short-term evaluation should not run beyond 6–9 administrations over 2–3 weeks without a documented response.

Oral ketamine should not be dispensed in large take-home quantities at the outset. Swainson et al. (23) underscored the value of cautious prescribing for non-parenteral ketamine, including treatment agreements, limited dispensing, and monitoring for craving, drug liking, and early-refill requests. Wherever possible, the first dose, and ideally the whole acute course, should be given under supervision. A less supervised approach may follow only after a favorable supervised response, and only for carefully selected patients: no current SUD, no identified diversion risk, reliable follow-up, stable blood pressure, and documented adherence to monitoring.

### 5.3 Oral tablets, capsules, troches, lozenges, and investigational prolonged-release products

The phrase “ketamine tablet” is clinically imprecise. Immediate-release compounded tablets, capsules, troches, and lozenges, and investigational extended- or prolonged-release tablets, absorb differently and are not interchangeable. Clinicians should not convert doses across oral products without formulation-specific pharmacokinetic and clinical data.

Glue et al. (15) tested the R-107 extended-release tablet in TRD using an enriched design. Participants first took 120 mg daily for 5 days; day-8 responders were then randomized to placebo or to twice-weekly doses of 30, 60, 120, or 180 mg for 12 weeks. Only the 180 mg arm separated from placebo on the primary 13-week MADRS outcome, with a least-squares mean difference of −6.1 points, and relapse rates fell across a dose gradient from about 70.6% on placebo to 42.9% on 180 mg. The trial also reported minimal dissociation, sedation, and blood-pressure change. Because non-responders were excluded during the open-label enrichment phase, however, these results are best read as promising, formulation-specific evidence rather than as support for a general oral ketamine protocol.

Walter et al. (16) reported two randomized trials of KET01, another prolonged-release racemic ketamine tablet. In the phase 2 depression trial, 240 mg/day showed early, exploratory MADRS advantages over placebo at days 4 and 7, but the primary day-21 endpoint was not met. In a separate phase 1 comparison, KET01 caused far less acute dissociation than intranasal esketamine, with a mean maximum CADSS change of 0.7 versus 29.6. Findings like these clarify why prolonged-release formulations are being pursued, but they do not license unsupervised extrapolation to compounded oral ketamine.

Smith-Apeldoorn et al. (24) illustrated how much dose and individual titration matter. A fixed low-dose regimen of oral esketamine, 30 mg three times daily for 6 weeks, did not separate from placebo on the Hamilton Depression Rating Scale, whereas a subsequent open-label phase using individually titrated 0.5-3.0 mg/kg twice-weekly oral esketamine was associated with symptom reduction.

### 5.4 Subcutaneous racemic ketamine

Subcutaneous ketamine is a practical alternative to intravenous infusion: it needs less equipment and can be easier to deliver in psychiatric settings. Loo et al. (11) conducted the KADS study, a 4-week randomized, double-blind, active-controlled trial comparing subcutaneous racemic ketamine with midazolam. In the flexible-dose cohort, ketamine was given twice weekly, starting at 0.5 mg/kg and titrated by response and tolerability up to 0.9 mg/kg. Remission at the end of treatment was significantly higher with ketamine than with midazolam, whereas the earlier fixed 0.5 mg/kg cohort showed no such separation. Acute adverse effects generally settled within about 2 hours and, in the published report, required no medical intervention.

Earlier work on the subcutaneous route includes small trials and observational studies using racemic ketamine at roughly 0.1-0.5 mg/kg and subcutaneous esketamine at roughly 0.5-1.0 mg/kg, once or twice weekly. Cavenaghi et al. (25) concluded that the subcutaneous route is promising, while noting that the evidence base is still smaller than for intravenous ketamine.

For psychiatrists giving off-label, non-intravenous racemic ketamine under supervision, subcutaneous administration currently offers the clearest short-term dosing strategy. A practical regimen is 0.5 mg/kg subcutaneously twice weekly, reassessed after two weeks. If response is partial or absent and tolerability is good, the dose can rise in 0.1 mg/kg steps toward 0.9 mg/kg, in line with the flexible-dose KADS approach. Frail older adults, patients with cardiovascular risk, and those highly anxious about dissociation may warrant a lower first exposure, though clinicians should bear in mind that very low doses may simply be less effective.

Subcutaneous ketamine belongs in a setting able to monitor blood pressure, pulse, oxygen saturation where clinically indicated, level of consciousness, dissociation, nausea, anxiety, and emergence reactions. Observation for at least 2 hours is prudent, especially during initiation and dose escalation.

### 5.5 Nasal spray formulations

#### 5.5.1 Esketamine nasal spray

Esketamine nasal spray has the best-defined product-specific dosing and monitoring framework of the routes discussed here. Janssen Pharmaceuticals, Inc. (6) gives the recommended TRD regimen for adults as 56 mg or 84 mg twice weekly during weeks 1-4, then 56 mg or 84 mg once weekly during weeks 5-8, and thereafter once weekly or every two weeks according to response and tolerability. For depressive symptoms in adults with MDD and acute suicidal ideation or behavior, the labeled dose is 84 mg twice weekly for 4 weeks, reduced to 56 mg if tolerability requires, and always alongside an oral antidepressant. For TRD, the same label directs clinicians to evaluate therapeutic benefit after the 4-week induction phase.

Esketamine is delivered from single-use devices of 28 mg each, so a 56 mg dose uses two devices and an 84 mg dose three. Janssen Pharmaceuticals, Inc. (6) advises a 5-minute rest between devices, treatment under clinical supervision, at least 2 hours of post-dose monitoring, and no driving or operating of hazardous machinery until the next day after a restful night’s sleep. Fasting precautions apply as well: no food for at least 2 hours and no liquids for at least 30 minutes before administration.

The esketamine trial program supplies concrete clinical benchmarks. Popova et al. (10) showed that flexibly dosed esketamine nasal spray, added to a newly started oral antidepressant, improved depressive symptoms in adults with TRD over a 4-week induction. Daly et al. (9) then showed that continued esketamine plus an oral antidepressant delayed relapse among stable remitters and stable responders in a randomized-withdrawal design. More recently, Janik et al. (7) found that esketamine monotherapy at 56 mg or 84 mg twice weekly improved MADRS scores relative to placebo at day 28, with separation apparent as early as 24 hours after the first dose, in a 378-participant phase 4 trial.

Where esketamine is available, clinicians should follow the approved label rather than adapt compounded ketamine protocols to it. The labeled schedule can also serve as a reference point for monitoring intensity when off-label nasal racemic ketamine is used, but the two products should never be treated as interchangeable.

#### 5.5.2 Compounded racemic ketamine nasal spray

Racemic intranasal ketamine should not be equated with esketamine nasal spray, because its evidence base is both limited and heterogeneous. An et al. (17) reviewed randomized, double-blind, placebo-controlled trials of intranasal ketamine or esketamine for depression and found that most of the randomized evidence concerned esketamine, with only a single randomized trial of racemic intranasal ketamine included. They described rapid-onset antidepressant effects and generally transient dissociation, but nothing amounting to a standardized racemic intranasal dosing model. Boudieu et al. (18) reached a similar conclusion, judging intranasal ketamine and esketamine clinically interesting for TRD and acute suicidal ideation while emphasizing that optimal dosing, frequency, comparative efficacy, and longer-term safety all still require study.

Compounded racemic intranasal ketamine should therefore be confined to settings with appropriate expertise, careful documentation, and no better route available. A cautious observed starting dose is 25–50 mg total racemic ketamine, delivered in divided, metered sprays over several minutes. Escalation to a total of 50–100 mg may be considered only if the initial dose is tolerated and the clinical response is inadequate. Frequency should generally be once or twice weekly during a short trial, with discontinuation if no meaningful improvement appears after 2–4 administrations.

Home bottles of compounded nasal ketamine invite dose imprecision, overuse, contamination, and diversion. If any take-home nasal product is used at all, it should come with limited dispensing, locked-storage counseling, no early refills, a written treatment agreement, monitoring for craving or drug liking, and objective symptom follow-up.

## 6 Patient selection

Suitable candidates are adults with a carefully confirmed depressive disorder, clinically significant current symptoms, an inadequate response to standard treatment, and a clear rationale for rapid intervention. Before offering ketamine, the psychiatrist should revisit the diagnosis, the adequacy of prior treatments, adherence, access to psychotherapy, substance use, personality and trauma-related contributors, sleep and pain disorders, medication interactions, and any medical contributors to the depression. A structured baseline assessment is summarized in **Table 4**, and suggested informed-consent elements are provided in (**Supplementary Material 2**; **Supplementary Table 1**).

**Table 4:** Baseline assessment before a short-term ketamine course.

Domain	Best-practice elements
Diagnostic confirmation	Confirm major depressive disorder or another depressive-disorder diagnosis, and reassess bipolarity risk, trauma, personality pathology, psychotic symptoms, substance-induced symptoms, and endocrine or neurologic contributors.
Treatment resistance	Document prior treatments, doses, durations, and adherence, along with psychotherapy, augmentation, any consideration of ECT or transcranial magnetic stimulation, and the patient-specific rationale for rapid intervention.
Risk assessment	Assess suicidal ideation, self-harm history, access to lethal means, protective factors, the crisis plan, and any need for a higher level of care.
Bipolarity and psychosis	Screen for mania, hypomania, mixed states, psychosis, a family history of bipolar disorder, and antidepressant-induced activation.
Substance use and diversion	Assess use of alcohol, cannabis, stimulants, sedatives, opioids, and hallucinogens, together with prior ketamine use, craving, drug liking, and diversion risk. Consider toxicology testing when clinically indicated.
Medical review	Measure blood pressure and pulse, and review cardiovascular, cerebrovascular, hepatic, urinary, respiratory, neurologic, and pregnancy status.
Medication review	Review sedatives, stimulants, antihypertensives, monoamine oxidase inhibitors, opioids, alcohol use, and any drugs affecting cognition or respiratory status.
Measurement baseline	Record MADRS, QIDS-SR, PHQ-9, CGI, and C-SSRS, adding YMRS where relevant and CADSS or Modified Observer’s Assessment of Alertness/Sedation Scale where available, together with a functional baseline.
Consent and logistics	Document off-label status where applicable, alternatives, expected benefits, uncertain durability, adverse effects, abuse risk, the escort and no-driving arrangements, and the stopping rules.

Relative or absolute exclusions include uncontrolled hypertension, unstable cardiovascular disease, recent myocardial infarction or stroke, aneurysmal vascular disease, arteriovenous malformation, active psychosis, current mania or a mixed state, uncontrolled SUD, high diversion risk, pregnancy unless a specialist risk-benefit review supports treatment, inability to give informed consent, lack of an escort after supervised sessions, and inability to comply with monitoring. Janssen Pharmaceuticals, Inc. (6) specifically contraindicates esketamine in patients with aneurysmal vascular disease or arteriovenous malformation, a history of intracerebral hemorrhage, or hypersensitivity to esketamine, ketamine, or excipients.

A SUD is not an automatic lifetime contraindication, but active, uncontrolled use, intoxication, repeated non-medical ketamine exposure, or a high diversion risk generally argues against treatment outside a specialist addiction-psychiatry framework. Most pivotal trials excluded active SUD, which limits certainty. Preliminary real-world data in 26 patients with TRD and comorbid SUD found symptom improvement without observed misuse during short follow-up, but this evidence is insufficient to override individualized risk assessment (26). Patients with a remote or stable SUD may be considered when the expected benefit is substantial, the disorder is actively treated, administration is supervised, take-home supply is avoided, and craving, intoxication, relapse, early-refill requests, and urine toxicology are monitored when clinically appropriate.

## 7 Treatment setting, prescriber responsibility, and supervision

Ketamine treatment should be matched to the clinical acuity and the facility’s capacity rather than offered as a setting-neutral prescription. The clinician who assumes responsibility should be legally authorized to prescribe the product and competent to diagnose and treat mood disorders, assess suicide and substance-use risk, recognize mania and psychosis, and manage route-specific adverse effects. In most psychiatric indications, a psychiatrist should lead or formally supervise treatment. A general physician may participate where local law permits and where there is documented psychiatric diagnostic input, a shared protocol, and immediate access to escalation; independent prescribing without mood-disorder expertise is not recommended for complex TRD, active suicidality, psychosis risk, or SUD.

In a specialist outpatient clinic or private practice, treatment is appropriate only when the service can provide baseline medical and psychiatric assessment, controlled-drug governance, trained staff, vital-sign monitoring, at least two hours of observation when indicated by route or label, emergency procedures, an escort/no-driving plan, and rapid psychiatric follow-up. Private practice should meet the same safety threshold as a hospital clinic; a less institutional environment does not justify less monitoring.

In inpatient hospital care, ketamine may be useful when severe depression, functional collapse, or suicidality requires continuous containment and rapid treatment. The inpatient unit can coordinate medication changes, suicide precautions, ECT comparison, and discharge planning, but administration should still occur in a quiet, appropriately equipped area rather than in the general ward milieu (21). Acute suicidality that requires a higher level of observation is an indication for inpatient care, not for unsupervised outpatient or take-home dosing.

Residential facilities, supported housing, nursing homes, and other non-medical psychiatric accommodation should not initiate or independently administer ketamine unless they are licensed and staffed to the same clinical standard as an outpatient treatment service. Housing staff may support transport, adherence, and post-treatment observation, but should not substitute for the prescribing clinician or trained healthcare personnel during administration. First doses and dose increases should always be supervised; self-administration is acceptable only where explicitly permitted by the approved product framework or an exceptional, documented off-label plan after demonstrated stability, and it should not be used in acute, high-risk, bipolar, psychotic, cognitively impaired, or substance-use-risk presentations.

## 8 Day-of-treatment protocol

Patients should be told not to drive to or from treatment and to arrange an escort, and to avoid alcohol, cannabis, and non-prescribed sedatives. Food and liquid restrictions may reduce nausea and are written explicitly into esketamine labeling; Janssen Pharmaceuticals, Inc. (6) advises no food for at least 2 hours and no liquids for at least 30 minutes beforehand. Similar precautions are reasonable for off-label formulations.

Before administration, staff should confirm identity, dose, route, and consent, and review interval clinical events, medication changes, sleep, substance use, suicidal ideation, and manic or psychotic symptoms, alongside baseline vital signs. A session should be postponed when blood pressure is clinically unsafe, when the patient is intoxicated, when an acute medical illness is present, when suicidality calls for a higher level of care, or when manic or psychotic symptoms have emerged.

During administration the setting should be quiet, low in stimulation, and medically supervised. Blood pressure and pulse should be taken at baseline and periodically through the acute psychoactive window; a common schedule is baseline, 30–40 minutes, 60 minutes, 90 minutes, and discharge, with extra checks if symptoms or blood-pressure elevations arise. Staff should watch for dissociation, anxiety, panic, sedation, nausea, vomiting, perceptual disturbance, agitation, and emergence reactions.

Discharge should wait until the patient is alert, oriented, ambulatory at baseline, clinically stable, and accompanied. Written post-treatment instructions should prohibit driving, operating machinery, signing major legal or financial documents, consuming alcohol, using recreational substances, and unsupervised sedative use until the following day. A minimum treatment-session safety checklist is summarized in **Box 1**.

Box 1 **Minimum treatment-session safety checklist**

1. Patient identity, route, dose, consent, and formulation verified.

2. No intoxication, new mania, psychosis, or acute medical instability.

3. Baseline BP and pulse acceptable under local protocol.

4. Escort and no-driving plan confirmed.

5. Emergency response process available; staff know escalation pathway.

6. Vitals and mental status monitored during the acute psychoactive window.

7. Discharge only when clinically stable and accompanied.

## 9 Monitoring response and stopping rules

A short-term ketamine intervention needs continuation and stopping rules set in advance. Response should be assessed with a standardized depression scale and a global clinical judgment before each dose, and suicidal ideation should be assessed at every encounter.

A workable stopping rule is to discontinue after 4–6 administrations when there is no meaningful improvement-no clear global clinical change and less than roughly 20-30% reduction in depressive symptom scores. If the patient reaches response (commonly defined as at least a 50% reduction) or remission, the acute course should close with a documented continuation plan rather than sliding into indefinite ketamine treatment.

Treatment should be withheld or stopped for sustained hypertension; severe or prolonged dissociation; psychosis; mania or hypomania; escalating drug-seeking behavior; cognitive deterioration; urinary symptoms suggestive of cystitis; an inability to follow safety instructions; or repeated requests for escalation without objective benefit. Short et al. (27) noted that psychiatric, psychotomimetic, cardiovascular, neurological, and gastrointestinal adverse effects are common in the acute ketamine literature, while the longer-term urological, cognitive, hepatic, and dependence risks demand particular vigilance with repeated exposure. The principal practical stopping rules are summarized in **Box 2**.

Box 2 **Practical stopping rules**

1. Non-response. Stop after 4–6 administrations when there is no meaningful clinical improvement.

2. Partial response without safety concern. Consider cautious route-appropriate titration, but only within the predefined dose range.

3. Response or remission. End the acute course and implement continuation treatment; do not default to indefinite ketamine.

4. Safety signal. Hold or discontinue for mania, psychosis, sustained hypertension, serious dissociation, prolonged sedation, cognitive deterioration, urinary symptoms, or misuse/diversion concerns.

5. Logistics failure. Hold treatment if the patient lacks an escort, cannot comply with post-treatment restrictions, or cannot participate in monitoring.

## 10 Frequency of administration

For short-term treatment, frequency has to balance rapid symptom relief against cumulative exposure and misuse risk. For subcutaneous racemic ketamine, the strongest current evidence supports twice-weekly dosing over 4 weeks with response-guided titration (11). For oral racemic ketamine solution, Domany et al. (12) used three doses per week for 21 days, though the broader review evidence is more heterogeneous and of lower certainty (13, 14). For esketamine nasal spray, frequency should follow the label: twice-weekly induction, then reduced frequency guided by response and tolerability (6).

Daily off-label ketamine should generally be avoided outside research protocols or formulation-specific evidence. If symptoms return quickly after a short course, the clinician should reassess the diagnosis, comorbidities, suicide risk, psychosocial precipitants, and alternative evidence-based treatments rather than simply pushing the dose or frequency higher.

## 11 Managing adverse effects

Common acute effects include dissociation, perceptual disturbance, dizziness, sedation, nausea, vomiting, anxiety, transient blood-pressure elevation, and impaired coordination. Janssen Pharmaceuticals, Inc. (6) calls for post-dose monitoring after esketamine on account of sedation, dissociation, respiratory depression, blood-pressure rises, and abuse or misuse risk. Systematic-review data for intranasal ketamine and esketamine describe dissociation and other effects as generally transient in randomized trials, while noting that long-term and route-specific safety are still incompletely defined (17, 18).

Management should lean first on reassurance, environmental control, vital-sign monitoring, and dose reduction at later sessions. Severe agitation, chest pain, neurological symptoms, syncope, severe hypertension, oxygen desaturation, or prolonged altered mental status all warrant medical evaluation. Benzodiazepines should not be given routinely before ketamine, since they may deepen sedation and could in theory blunt the psychological or antidepressant effects; where severe anxiety or agitation calls for them, they should be used judiciously with respiratory and sedation monitoring.

## 12 Misuse, diversion, and controlled-substance governance

Ketamine carries abuse potential, and the risk rises when treatment is unsupervised, dosing is frequent, dispensed quantities are large, or patients have active SUDs. Swainson et al. (23) recommended cautious prescribing of non-parenteral ketamine, written treatment agreements, limited dispensing, monitoring for craving or drug liking, scrutiny of early-refill requests, and surveillance for urinary symptoms.

The U.S. Food and Drug Administration (5) warned that compounded ketamine products, oral formulations included, are not FDA-approved for psychiatric disorders and are not assessed for safety, effectiveness, or quality before marketing. The same communication stressed that at-home use without onsite monitoring may heighten risk from sedation, dissociation, vital-sign changes, abuse and misuse, psychiatric events, respiratory depression, and lower urinary tract or bladder symptoms. Regulatory requirements vary between jurisdictions, but the underlying principle holds everywhere: convenience should not be bought at the price of adequate monitoring.

Best practice includes a written controlled-substance protocol, secure storage, dose logs, inventory reconciliation, prescription-monitoring checks where available, no replacement of lost medication, no early refills, and explicit discontinuation criteria. Jelen et al. (4) argued for guidance, systematic monitoring, and registry-level data as ketamine practice moves beyond research settings. For oral and nasal formulations, the first dose and every escalation should be observed whenever possible, and less-supervised treatment should be reserved for carefully selected patients who have shown a favorable response and good tolerability under supervision, carry no misuse or diversion risk, can engage in reliable follow-up, have stable cardiovascular status, and genuinely cannot access supervised care. The principal controlled-substance safeguards are summarized in **Table 5**.

**Table 5:** Controlled-substance safeguards for off-label racemic ketamine.

Phase	Safeguards
Before treatment	Document the diagnosis, treatment resistance, rationale, alternatives, and off-label consent, together with substance-use history, diversion risk, baseline urinary symptoms, and a prescription-monitoring review where available.
During acute treatment	Observe the first dose and every increase, and dispense only the minimum quantity required. Monitor for craving, drug liking, intoxication, early-refill requests, reports of lost medication, intoxicated attendance, and requests for unsupervised escalation.
For any take-home dosing	Use a written treatment agreement, require locked storage, and prohibit sharing, selling, or self-directed dose changes. Require dose logs, schedule frequent reviews, do not replace lost supply, and avoid bulk dispensing.
When risk emerges	Hold or stop ketamine and reassess for a substance-use disorder. Consider toxicology testing, collateral information, revision of the treatment contract, or referral to addiction services.
For repeated or maintenance exposure	Add cognitive screening and urinary-symptom surveillance, and periodically reassess whether ketamine remains necessary and whether a lower-frequency strategy is feasible.

## 13 Practical route selection

Subcutaneous ketamine may be the preferred off-label route for racemic ketamine when supervised treatment and appropriate monitoring are available but intravenous infusion is not feasible. It offers a well-characterized short-term dosing strategy, is relatively simple to give, and is backed by randomized evidence that flexible dose titration can be implemented effectively (11). A practical pathway for supervised off-label racemic ketamine is provided in Algorithm A.

Algorithm A **Supervised off-label racemic ketamine when rapid non-IV treatment is needed.**

1. Confirm diagnosis, treatment resistance, medical suitability, and consent.
2. Prefer subcutaneous racemic ketamine if supervised clinic dosing is feasible.
3. Start at 0.5 mg/kg subcutaneously.
4. Treat twice weekly.
5. Reassess after 4 administrations.
6. If partial response and good tolerability, titrate by approximately 0.1 mg/kg toward 0.9 mg/kg.
7. Stop after 4–6 administrations if no meaningful benefit.
8. Stop or reduce dose for clinically significant adverse effects.
9. End the acute course after response/remission and transition to continuation care.

An oral ketamine solution may be considered where supervised parenteral treatment or regulated esketamine is unavailable. Here the clinician has to weigh its slower onset and more variable pharmacokinetics, the comparatively limited evidence, and the greater diversion potential once medication goes home (8, 13, 23). A practical pathway for oral ketamine when parenteral or labeled nasal treatment is inaccessible is provided in Algorithm B.

Algorithm B **Oral ketamine when parenteral or labeled nasal treatment is inaccessible.**

1. Prefer oral solution over nonstandard tablets for dose accuracy.
2. Start 0.5-1.0 mg/kg orally under observation.
3. Dose 2–3 times weekly for a short trial.
4. Increase by 0.25-0.5 mg/kg only if tolerated and inadequate response.
5. Reassess after 4–6 administrations.
6. Avoid large take-home supplies.
7. Use treatment agreement, limited dispensing, and misuse monitoring.
8. Stop if no meaningful benefit, misuse concerns, urinary symptoms, mania, psychosis, or clinically significant cardiovascular effects emerge.

Oral ketamine tablets likewise call for caution, since formulation differences can carry real clinical consequences. Glue et al. (15) and Walter et al. (16) reported that specialized prolonged-release tablets may soften the acute dissociative and hemodynamic effects. Esketamine nasal spray should be used according to the approved label and, where available and clinically appropriate, is preferable to compounded racemic nasal spray (6). Compounded racemic nasal ketamine is best kept as a last-line non-intravenous option, because dosing precision, mucosal absorption, misuse potential, and the overall quality of the supporting evidence all compare unfavorably with regulated esketamine (17, 18). A practical pathway for nasal treatment is provided in Algorithm C.

Algorithm C **Nasal treatment.**

1. Use labeled esketamine where available and appropriate.
2. Follow product-specific dosing, fasting, administration, monitoring, and driving restrictions.
3. For compounded racemic nasal ketamine, restrict use to specialist settings.
4. Start 25–50 mg total intranasal racemic ketamine under observation.
5. Consider cautious titration only with documented tolerability.
6. Avoid unsupervised bottles during acute initiation.
7. Stop early if there is no objective benefit.

## 14 Discussion

The field is steadily moving from proving that ketamine has antidepressant effects to working out how to deliver them-questions of route, dose, frequency, patient selection, monitoring, treatment duration, and clinical governance. On current evidence, a conservative hierarchy is warranted. Labeled esketamine has the clearest regulatory structure and monitoring framework. Among the off-label, non-intravenous racemic approaches, subcutaneous ketamine has the strongest short-term dosing evidence. Oral ketamine remains promising but constrained by variable bioavailability, heterogeneous dosing practice, product-quality concerns, and diversion risk. Compounded racemic nasal ketamine should not be treated as equivalent to esketamine and should be used only under robust safeguards.

A central practical point is that ketamine response should be measured, not assumed. Without standardized symptom scales and predefined stopping rules, clinicians can drift into repeated exposure despite uncertain ongoing benefit; this is a real concern, since acute tolerability does not establish long-term safety, and non-parenteral formulations may open more room for misuse and diversion. Beaglehole et al. (28) highlighted the value of multidisciplinary, service-level guidance for specialist ketamine services, while Jelen et al. (4) pressed the case for registries and systematic monitoring as clinical use widens.

The oral-ketamine literature shows especially clearly why formulation specificity matters. Domany et al. (12) points to a possible benefit from repeated oral ketamine; Glue et al. (15) and Walter et al. (16) suggest that specialized prolonged-release tablets may lower the acute dissociative and hemodynamic burden; and Smith-Apeldoorn et al. (24) shows that fixed low-dose oral esketamine can fail even where individually titrated open-label dosing looks clinically active. These findings are complementary, not interchangeable.

This manuscript deliberately concentrates on acute intervention rather than maintenance. Maintenance ketamine therapy sits outside the primary scope, because the durability of response, cumulative exposure, urinary and cognitive effects, optimal intervals, and discontinuation strategies remain insufficiently defined across formulations. Where maintenance is contemplated, it should involve a separate consent process, periodic attempts to reduce frequency, and clear documentation that benefits continue to outweigh risks.

### 14.1 Forensic hospitals and prisons

Forensic psychiatric hospitals and prisons should not be excluded categorically, because equivalence of healthcare requires access to effective treatment for severe depression. However, the evidence specific to detained populations is currently extremely limited and is largely ethical, legal, and practice-oriented rather than trial-based (29). Use in these settings should therefore be exceptional, specialist-led, and fully supervised, with the same diagnostic and physiological safeguards as hospital treatment plus enhanced attention to coercion, voluntariness of consent, confidentiality, security, trafficking or diversion, drug-related debts, and continuity after transfer or release.

A secure setting may reduce unsupervised access to the drug but does not remove misuse risk. Medication should remain under institutional control, administration should be directly observed, and no take-home supply should be provided. The decision should be clinically independent of disciplinary or custodial objectives, and refusal should not carry adverse custodial consequences. Where emergency transfer, medical monitoring, or psychiatric follow-up cannot be guaranteed, ketamine should not be initiated.

## 15 Limitations

This is a narrative review with practical recommendations, not a systematic review or a formal clinical guideline. The strength and quality of the evidence vary considerably across routes and formulations, and many studies of off-label racemic ketamine are limited by small samples, heterogeneous methods, short follow-up, or reliance on extrapolation from intravenous data. In every case, local legislation, controlled-substance regulations, professional standards, and approved product labeling may differ by jurisdiction and should take precedence over any general recommendation offered here. The literature search was purposive rather than systematic, the stated source count is not a PRISMA study-flow denominator, and the evidence categories are a transparent narrative framework rather than a validated grading instrument. Several setting-specific and operational recommendations necessarily rely on consensus guidance and conservative expert judgment.

## 16 Conclusions

Short-term ketamine treatment for depressive disorders is best understood as a structured psychiatric intervention rather than a simple prescription. Good practice is diagnosis-driven, measurement-based, supervised, and time-limited, with explicit criteria for dose escalation and discontinuation. Among off-label non-intravenous options, supervised subcutaneous racemic ketamine is currently the best-supported route. Oral ketamine can suit carefully selected patients but demands attention to formulation, dispensing, monitoring, and misuse risk. Esketamine nasal spray should be used according to the approved label, while compounded racemic intranasal ketamine should be reserved for exceptional circumstances given its limited evidence and product variability.
