# Supplementary material for: Short-term ketamine interventions for depressive disorders: practical best-practice recommendations for psychiatrists using oral, subcutaneous, and nasal formulations
Source: Front Psychiatry. 2026 Sep 3;17:1931248. doi: 10.3389/fpsyt.2026.1931248 (PMC13584313; doi:10.3389/fpsyt.2026.1931248)
Supplement: Supplementary file 1 [file SupplementaryFile1.docx]

# Supplementary Material 1. PubMed/MEDLINE search strategy

**PubMed/MEDLINE core search string:**

((ketamine[MeSH Terms] OR ketamine[Title/Abstract] OR esketamine[Title/Abstract] OR racemic ketamine[Title/Abstract]) AND (treatment-resistant depression[Title/Abstract] OR TRD[Title/Abstract] OR major depressive disorder[Title/Abstract] OR bipolar depression[Title/Abstract]) AND (oral[Title/Abstract] OR tablet[Title/Abstract] OR capsule[Title/Abstract] OR troche[Title/Abstract] OR lozenge[Title/Abstract] OR solution[Title/Abstract] OR subcutaneous[Title/Abstract] OR intranasal[Title/Abstract] OR nasal[Title/Abstract] OR dose[Title/Abstract] OR dosing[Title/Abstract] OR frequency[Title/Abstract] OR administration[Title/Abstract] OR monitoring[Title/Abstract] OR misuse[Title/Abstract] OR diversion[Title/Abstract]))

# Supplementary Material 2. Suggested informed-consent elements for off-label racemic ketamine

Supplementary Table 1. Suggested informed-consent language

| **Consent element** | **Suggested language** |
| --- | --- |
| Off-label status | State that oral, subcutaneous, or compounded racemic nasal ketamine is off-label for depressive disorders unless local authorization differs. |
| Expected benefit | Possible rapid reduction in depressive symptoms in responders; uncertain durability; not guaranteed remission; not a substitute for comprehensive psychiatric care. |
| Alternatives | Medication optimization, psychotherapy, ECT, TMS, lithium or antipsychotic augmentation, hospitalization, intensive outpatient treatment, and labeled esketamine where available. |
| Acute risks | Dissociation, perceptual disturbance, anxiety, sedation, nausea, vomiting, dizziness, blood-pressure elevation, impaired coordination, and transient cognitive impairment. |
| Longer-term and cumulative risks | Uncertain optimal duration; possible cognitive, urinary, hepatic, tolerance, and misuse/diversion concerns with repeated exposure. |
| Safety restrictions | No driving or hazardous work until the next day after restful sleep; escort required after supervised sessions; no alcohol, recreational substances, or unsupervised sedatives. |
| Stopping rules | Discontinue for non-response, mania, psychosis, sustained hypertension, severe dissociation, urinary symptoms, misuse risk, or non-adherence to safety requirements. |
